# Sex-split analysis of pathology and motor-behavioral outcomes in a mouse model of CLN8-Batten disease reveals an increased disease burden and trajectory in female *Cln8*^*mnd*^ mice

**DOI:** 10.1186/s13023-022-02564-7

**Published:** 2022-11-11

**Authors:** Andrew D. Holmes, Katherine A. White, Melissa A. Pratt, Tyler B. Johnson, Shibi Likhite, Kathrin Meyer, Jill M. Weimer

**Affiliations:** 1 Pediatrics and Rare Diseases Group, Sanford Research, 2301 E 60Th St N, Sioux Falls, SD USA; 2 Department of Pediatrics, Sanford School of Medicine, University of South Dakota, Sioux Falls, SD USA; 3 The Research Institute at Nationwide Children’s Hospital, Columbus, OH USA; 4 Department of Pediatrics, The Ohio State University, Columbus, OH USA

**Keywords:** CLN8, Batten disease, Sex differences, Lysosomal storage disorders, Disease progression, AAV9 gene therapy

## Abstract

**Background:**

CLN8-Batten disease (CLN8 disease) is a rare neurodegenerative disorder characterized phenotypically by progressive deterioration of motor and cognitive abilities, visual symptoms, epileptic seizures, and premature death. Mutations in *CLN8* results in characteristic Batten disease symptoms and brain-wide pathology including accumulation of lysosomal storage material, gliosis, and neurodegeneration. Recent investigations of other subforms of Batten disease (CLN1, CLN3, CLN6) have emphasized the influence of biological sex on disease and treatment outcomes; however, little is known about sex differences in the CLN8 subtype. To determine the impact of sex on CLN8 disease burden and progression, we utilized a *Cln8*^*mnd*^ mouse model to measure the impact and progression of histopathological and behavioral outcomes between sexes.

**Results:**

Several notable sex differences were observed in the presentation of brain pathology, including *Cln8*^*mnd*^ female mice consistently presenting with greater GFAP^+^ astrocytosis and CD68^+^ microgliosis in the somatosensory cortex, ventral posteromedial/ventral posterolateral nuclei of the thalamus, striatum, and hippocampus when compared to *Cln8*^*mnd*^ male mice. Furthermore, sex differences in motor-behavioral assessments revealed *Cln8*^*mnd*^ female mice experience poorer motor performance and earlier death than their male counterparts. *Cln8*^*mnd*^ mice treated with an AAV9-mediated gene therapy were also examined to assess sex differences on therapeutics outcomes, which revealed no appreciable differences between the sexes when responding to the therapy.

**Conclusions:**

Taken together, our results provide further evidence of biologic sex as a modifier of Batten disease progression and outcome, thus warranting consideration when conducting investigations and monitoring therapeutic impact.

**Supplementary Information:**

The online version contains supplementary material available at 10.1186/s13023-022-02564-7.

## Background

Neuronal ceroid lipofuscinoses (NCLs) are a family of inherited lysosomal diseases that result in neurodegenerative disease within pediatric and adult populations. Commonly known as Batten disease, NCLs have an extensive range of phenotypic presentation, although most forms can be clinically characterized by declining cognitive and motor functions, ocular dysfunction, and eventual blindness, epilepsy, and a decreased lifespan [1] (for a recent review see [2]). Although NCLs are considered rare in nature, together they are the most prevalent neurodegenerative disease in the pediatric population with an estimated incidence of 2–4/100,000 births [3, 4] and an even greater incidence within certain populations. The etiology of NCLs is due to a mutation in one of at least 13 currently identified ceroid lipofuscinosis neuronal (CLN) genes—often encoding enzymes or regulatory proteins involved in proper lysosomal function [5, 6]. One of these genes, *CLN8,* encodes a transmembrane endoplasmic reticulum (ER) protein (CLN8) that has been shown to be involved in the trafficking of lysosomal-destined enzymes between the ER and Golgi, in addition to integral involvement with other lysosomal processes such as biogenesis [6, 7]. Additionally, studies have demonstrated neuronal-specific roles of *CLN8* in neurite maturation, differentiation, and support of various neuronal populations [7–9]. Mutations in *CLN8* results in characteristic NCL symptoms and brain-wide pathology including accumulation of lysosomal storage material, gliosis, and other neurodegenerative signs [6, 10].

CLN8 Batten disease (CLN8 disease) is a variant late-infantile form of Batten disease with an onset of symptoms generally between 5 and 10 years old [11]. Patients with CLN8 disease present with progressive deterioration of motor and cognitive abilities, visual symptoms, and epileptic seizures [6]. Two classic variants arising from mutations of *CLN8* have been well described: (1) “Northern Epilepsy” is a condition characterized by epileptic seizures (tonic–clonic and/or complex partial) with peak frequency in adolescence followed by declining cognition and deteriorating motor skills due to cerebellar atrophy [12, 13]. Hirvasniemi et al. first identified Northern Epilepsy within patients of Northern Finland where patients all shared a homozygous missense mutation of *CLN8* [13], but this subtype has also been described to result from other mutations in other populations [14, 15]; (2) Variant Late-infantile NCL (vLINCL) is a more severe phenotype associated with *CLN8* mutation first identified in Turkish families. This variant typically presents as epileptic seizures, motor and cognitive deterioration**,** and visual disturbances (which help distinguish it from Northern Epilepsy clinically). Furthermore, patients with vLINCL experience more severe disease progression with motor and cognitive deterioration occurring within several years, as compared to Northern Epilepsy which progresses over several decades [16]. Despite these two well-described phenotypes of CLN8 disease within distinct populations, cases have been described in a multitude of geographic locations throughout the world with variability in disease progression [14, 15, 17–21]. As such, clinical presentation of CLN8 disease may not always fall into a discrete category and suspicion of the disorder warrants further genetic and diagnostic testing [16].

Recently, greater emphasis has been placed on understanding and identifying sex distinctions as an important modulator of physiology, anatomy, and pathology in disease, including within various forms of Batten disease [22–25]. A multitude of neurodegenerative diseases demonstrate sex biases, such as greater prevalence of Alzheimer’s disease in women and increased prevalence of Parkinson’s disease and amyotrophic lateral sclerosis in men [26]. The field of Batten disease is no different: NCLs have been shown to demonstrate sex-based clinic and pathologic differences in patients and in animal models. Although male subjects typically experience earlier disease onset, females with juvenile NCL (JNCL; CLN3 Disease) suffer a more rapid disease progression characterized by quicker cognitive decline, loss of motor coordination, and earlier death [27, 28]. Further, Cialone et al. [28] described female patients as having a poorer quality of life due to greater physical impairment. Overall, identifying sex differences (or lack thereof) in humans with Batten disease is exceedingly difficult due to various mutations within the range of *CLN* genes and complex interactions between their respective unique genetics and environment.

The utilization of murine models in Batten disease research has greatly expanded the ability to investigate sex differences in this family of diseases, in addition to highlighting the importance of sex as a factor to be considered when designing and analyzing therapeutic trials [29]. For instance, sex-dependent differences in gene expression response to galactosylceramide were found in the *Cln3*^*Δex7/8*^ murine model [30]. Further, Poppens et al. described female *Cln6*^*nclf*^ mice to experience accelerated disease progression, more severe behavioral issues and motor decline, and differences in histopathological effects [31]. A prior investigation of *Cln8*^*mnd*^ mice revealed sex differences in retinal vulnerability where female retinas exhibited higher oxidation rates and caspase-3 mediated apoptosis, in addition to a more severe histopathological profile of the retina [32]. However, the disease associated phenotypes in relationship to sex examined in this study were limited to visual deficits in the *Cln8*^*mnd*^ mouse model. To add to this body of work, we examined the influence of sex on psychomotor behavioral outcomes and histopathology within thalamus and primary somatosensory cortex of *Cln8*^*mnd*^ mice. Additionally, *Cln8*^*mnd*^ sexual dimorphisms in AAV9 gene therapy response were also explored.

## Results

### Cln8^mnd^ mice have sex dependent differences in storage material accumulation

The *Cln8*^*mnd*^ mouse model is a widely used and well-characterized mouse model of CLN8 disease, in which mice show disease associated histopathologic changes in the brain as early as 2 months of age, behavioral deficits by 6 months of age, and premature death by 10 months of age [6, 33]. Here, *Cln8*^*mnd*^ mice at varying ages were analyzed to determine whether sex differences existed in classic Batten disease pathologies within somatosensory thalamic nuclei (VPM/VPL) and the somatosensory cortex (S1BF), as well as lesser studied regions such as the striatum and CA3 of the hippocampus.

**Fig. 1:**
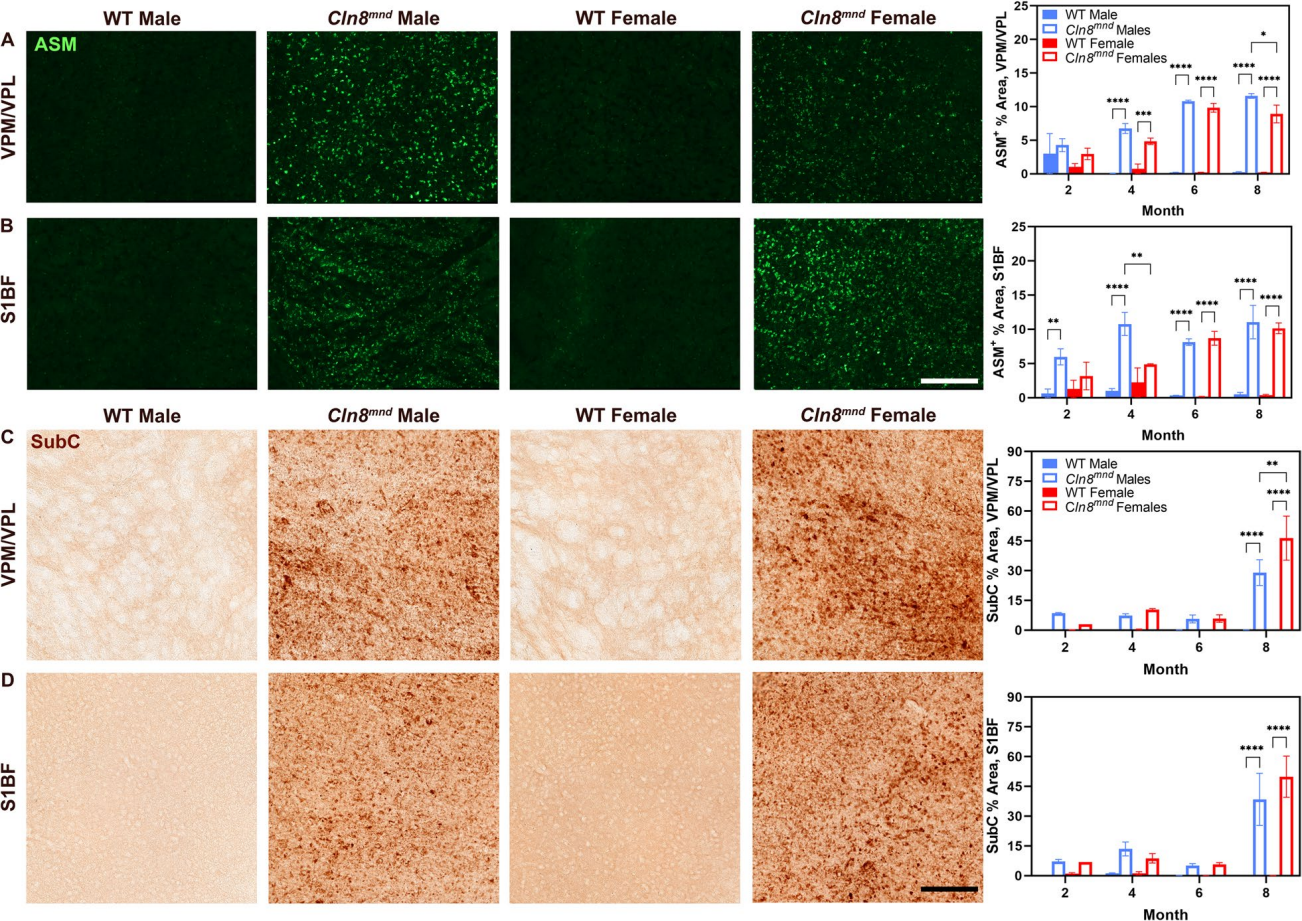
Sex differences evident in *Cln8*^*mnd*^ accumulation of autofluorescent storage material and ATP Synthase subunit C. *Cln8*^*mnd*^ males demonstrate greater ASM accumulation within the VPM/VPL at 8 months (**A**) and within the S1BF at 4 months of age (**B**). *Cln8*^*mnd*^ females show enhanced SubC accumulation at 8 months within the VPM/VPL (**C**) while no sex differences were detected in the S1BF (**D**). Two-way ANOVA with Fisher’s LSD post-hoc. Mean ± SEM, n = 2–4 animals/sex/group, detailed n described in Additional file 4: Table S1. *p < 0.05, **p < 0.01, ***p < 0.001, ****p < 0.0001. ASM Scale Bar: 200 µm; SubC Scale Bar: 150 µm

Autofluorescent storage material (ASM) accumulation is a pathological characteristic of all Batten disease variants, and *Cln8*^*mnd*^ mice had greater accumulation of ASM compared to wild type mice within both the VPM/VPL and S1BF at most time points studied (Fig. 1A, B). While there were generally no differences in ASM accumulation between the sexes in the VPM/VPL, *Cln8*^*mnd*^ males showed greater ASM accumulation than female counterparts at 8 months of age (Fig. 1A). Importantly, *Cln8*^*mnd*^ males showed earlier and more severe ASM accumulation in the S1BF than *Cln8*^*mnd*^ females, with ASM accumulation beginning at 2 months of age and showing a larger burden at 4 months of age (Fig. 1B). This male-specific difference disappeared at later time points, which may indicate that males have sooner pathological onset of ASM accumulation while females have a quicker progression of accumulation after 4 months of age.

Mitochondrial ATP synthase subunit c (SubC) is one of the known constituents of the storage material accumulated in various forms of Batten disease [34, 35]. *Cln8*^*mnd*^ mice had greater amounts of SubC accumulation relative to wild type mice at 8 months of age within both anatomic locations (Fig. 1C-D). While there were no differences in SubC accumulation between male and female *Cln8*^*mnd*^ mice for most time points, *Cln8*^*mnd*^ females had greater accumulation of SubC within the thalamic nuclei and striatum at 8 months of age relative to males (Fig. 1C, Additional file 1: Fig. S1A). Although some differences were observed at end-stage disease, ASM and SubC accumulation show little sex-dependent differences over the course of the disease.

### ***Cln8***^***mnd***^*** mice have female specific increases in astrocyte and microglial reactivity***

**Fig. 2:**
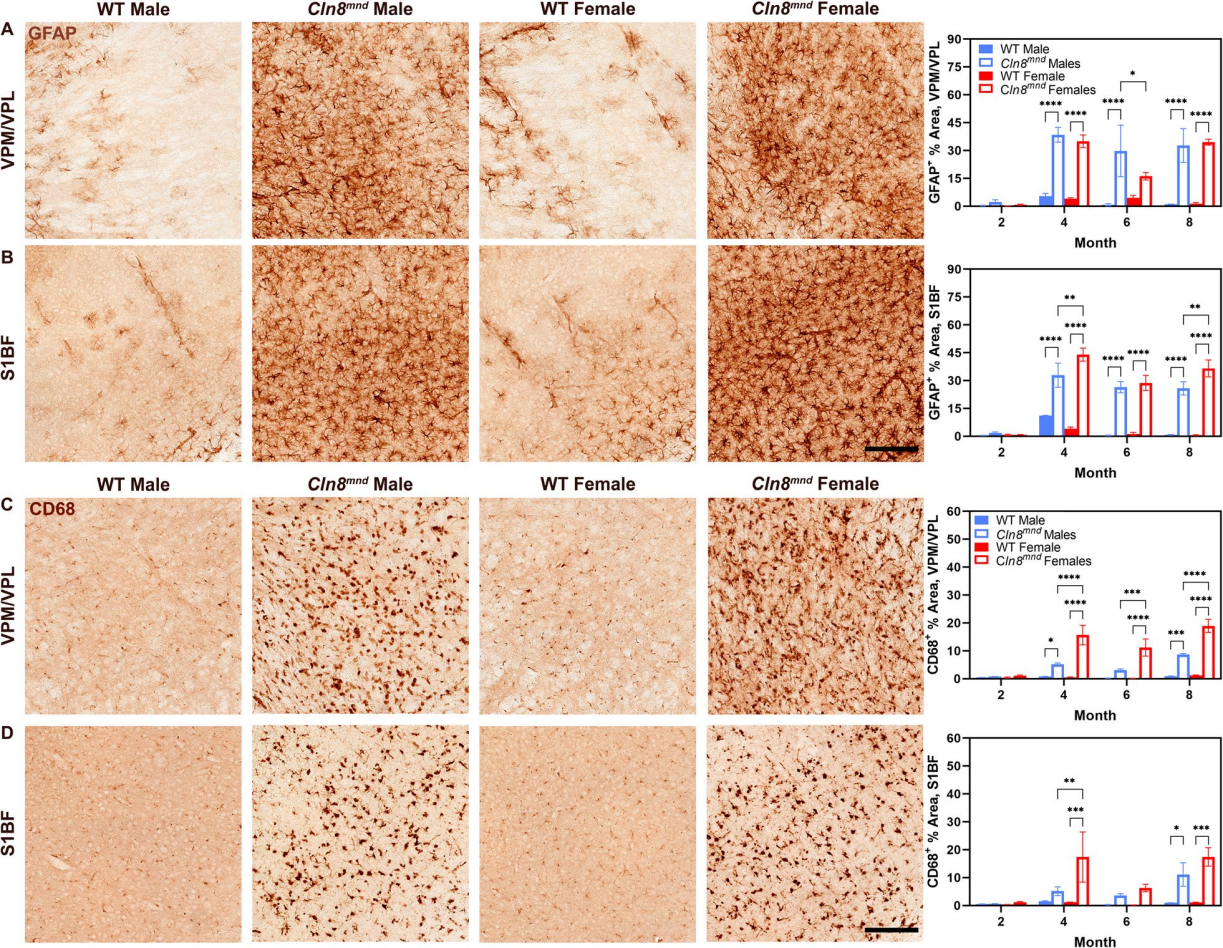
Female *Cln8*^*mnd*^ mice show enhanced glial activation in brain. Male *Cln8*^*mnd*^ mice demonstrate greater astrocyte expression (GFAP^+^) within the VPM/VPL of the thalamus at 6 months of age (**A**) whereas female *Cln8*^*mnd*^ mice have greater expression within the S1BF at 4 and 8 months of age (**B**). Female *Cln8*^*mnd*^ mice exhibit enhanced microglial activation (CD68^+^) at months 4, 6, and 8 within the VPM/VPL (**C**) and at month 4 within the S1BF (**D**). Two-way ANOVA with Fisher’s LSD post-hoc. Mean ± SEM, n = 1–4 animals/sex/group, detailed n described in Additional file 4: Table S1. *p < 0.05, **p < 0.01, ***p < 0.001, ****p < 0.0001. Scale Bars: 150 µm

Glial fibrillary acidic protein (GFAP) is an intermediate filament commonly associated with reactive astrocytes of the central nervous system (CNS) and it can be utilized to indicate non-specific pathological reactions [36, 37]. *Cln8*^*mnd*^ mice displayed increased evidence of GFAP^+^ astrocytosis compared to wild type mice at most time points in the VPM/VPL and S1BF (Fig. 2A, B). Interestingly, *Cln8*^*mnd*^ males had increased astrocytosis within the VPM/VPL at 6 months of age (Fig. 2A), yet *Cln8*^*mnd*^ females had greater evidence of astrocytosis within the somatosensory cortex at 4 and 8 months of age, and in the striatum at 8 months of age (Fig. 2B, Additional file 1: Fig. S1B). Astrocytic activation progresses with time and differs by sex and brain region.

Cluster of differentiation protein 68 (CD68) is a cell surface marker for microglial activation often used in mouse models of neurodegenerative disease [6, 31]. Akin to the astrocyte response, *Cln8*^*mnd*^ mice had enhanced microglial activation relative to wild type within both anatomic sites beginning at 4 months of age (Fig. 2C, D). Overall, within both the VPM/VPL and S1BF, there was a marked increase in reactive astrocytosis and microgliosis from 2 to 4 months of age. When analyzing by sex, *Cln8*^*mnd*^ females displayed substantially greater evidence of CD68^+^ microgliosis at 4, 6, and 8 months of age within the VPM/VPL (Fig. 2C) and at 4 months of age within the S1BF (Fig. 2D). Additionally, female *Cln8*^*mnd*^ mice showed exacerbated microgliosis in the striatum and CA3 of the hippocampus, prior to when their male counterparts present with a phenotype in these regions (Additional file 1: Fig. S1C). Taken together, female *Cln8*^*mnd*^ mice show a consistent upregulation of astrocyte and microglia reactivity in several regions of the brain that is more severe than their male counterparts.

Lastly, as these are models of a neurodegenerative disease, thinning of the cortical plate was measured at two time points to determine if cell death occurred in a sex specific manner. From this broad experiment, *Cln8*^*mnd*^ mice showed no cortical thinning at 2 or 6 months of age, regardless of sex (Additional file 2: Fig. S2).

### ***Cln8***^***mnd***^*** mice have sex dependent differences in life span and motor-behavioral assessments***

**Fig. 3:**
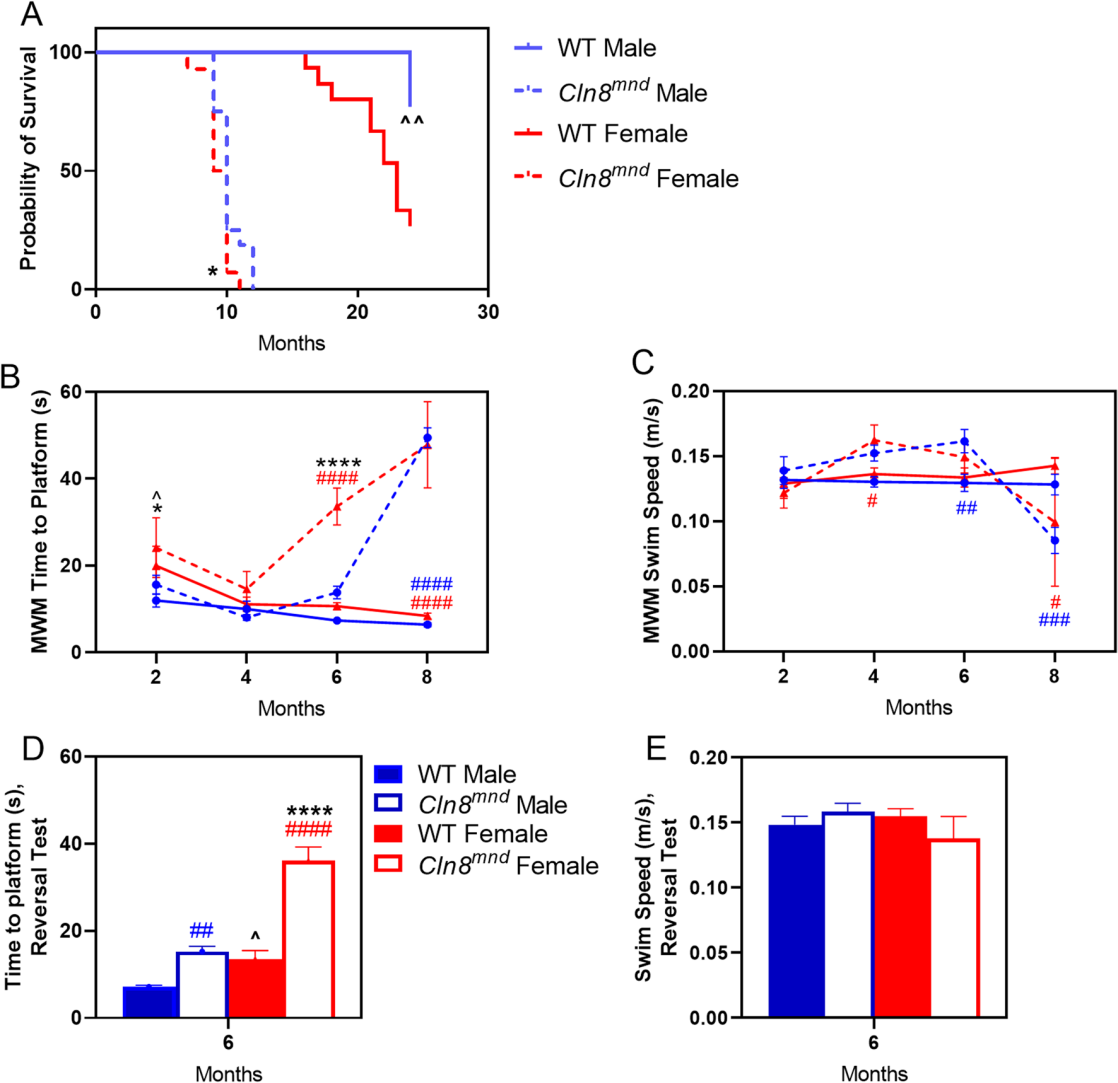
Sex dependent differences in *Cln8*^*mnd*^ life span and motor-behavioral assays. *Cln8*^*mnd*^ females have a decreased life span compared to *Cln8*^*mnd*^ males, with a median age of 9.5 months and 10 months respectively (**A**). Morris Water Maze (MWM) demonstrating *Cln8*^*mnd*^ females taking significantly longer to complete the task at 2 and 6 months of age when compared to *Cln8*^*mnd*^ males (**B**), of which was not accounted for by differing swim speed (**C**). *Cln8*^*mnd*^ females completed the reverse MWM in greater time compared to their male counterparts despite no difference in swim speeds (**D**, **E**). Comparisons of wild type males versus wild type females^^^, *Cln8*^*mnd*^ males versus *Cln8*^*mnd*^ females*, *Cln8*^*mnd*^ males versus wild type males^#^, and *Cln8*^*mnd*^ females versus wild type females^#^. Survival curve: log-rank (Mantel–Cox); n = 13–16 animals/sex. MWM: Two-way ANOVA with Fisher’s LSD post-hoc. Mean ± SEM, n = 2–11 animals/sex/group, detailed n described in Additional file 4: Table S1. *p < 0.05, **p < 0.01, ***p < 0.001, ****p < 0.0001

To determine if there were sex differences in *Cln8*^*mnd*^ survival and motor-behavioral performance, animals were examined at 2, 4, 6, 8, and 10 months of age for behavioral outcomes and through 24 months of age for survival assessment. As a whole, *Cln8*^*mnd*^ mice perished earlier than their respective wild type counterparts, with a median survival of 10 months of age (Fig. 3A). Importantly, *Cln8*^*mnd*^ females perished significantly earlier than their *Cln8*^*mnd*^ male counterparts, living approximately 0.5 months less compared to *Cln8*^*mnd*^ males (Fig. 3A).

Mice were examined in a Morris Water Maze (MWM) in which they were trained to find a hidden platform in a pool of water to assess vision, memory, and spatial learning. *Cln8*^*mnd*^ mice took significantly longer to complete the task compared to wild type mice, with *Cln8*^*mnd*^ females showing poor performance at 6 and 8 months of age and *Cln8*^*mnd*^ males showing poor performance at 8 months of age (Fig. 3B). *Cln8*^*mnd*^ female mice performed worse at an earlier stage than their male comparisons during MWM assessments. Specifically, *Cln8*^*mnd*^ males completed the MWM in a significantly shorter time compared to *Cln8*^*mnd*^ females at 2 and 6 months. Accounting for swim speed did not impact these results, indicating that *Cln8*^*mnd*^ females have greater MWM deficiencies than males of the same age (Fig. 3C). At 8 months of age, *Cln8*^*mnd*^ females and *Cln8*^*mnd*^ males had no observed difference. A reverse MWM assessment, where the hidden platform was moved to a novel location, was conducted when the mice were at 6 months of age, which demonstrated that *Cln8*^*mnd*^ females took significantly more time to complete the assessment than their *Cln8*^*mnd*^ male counterparts despite similar swim speed (Fig. 3D, E).

**Fig. 4:**
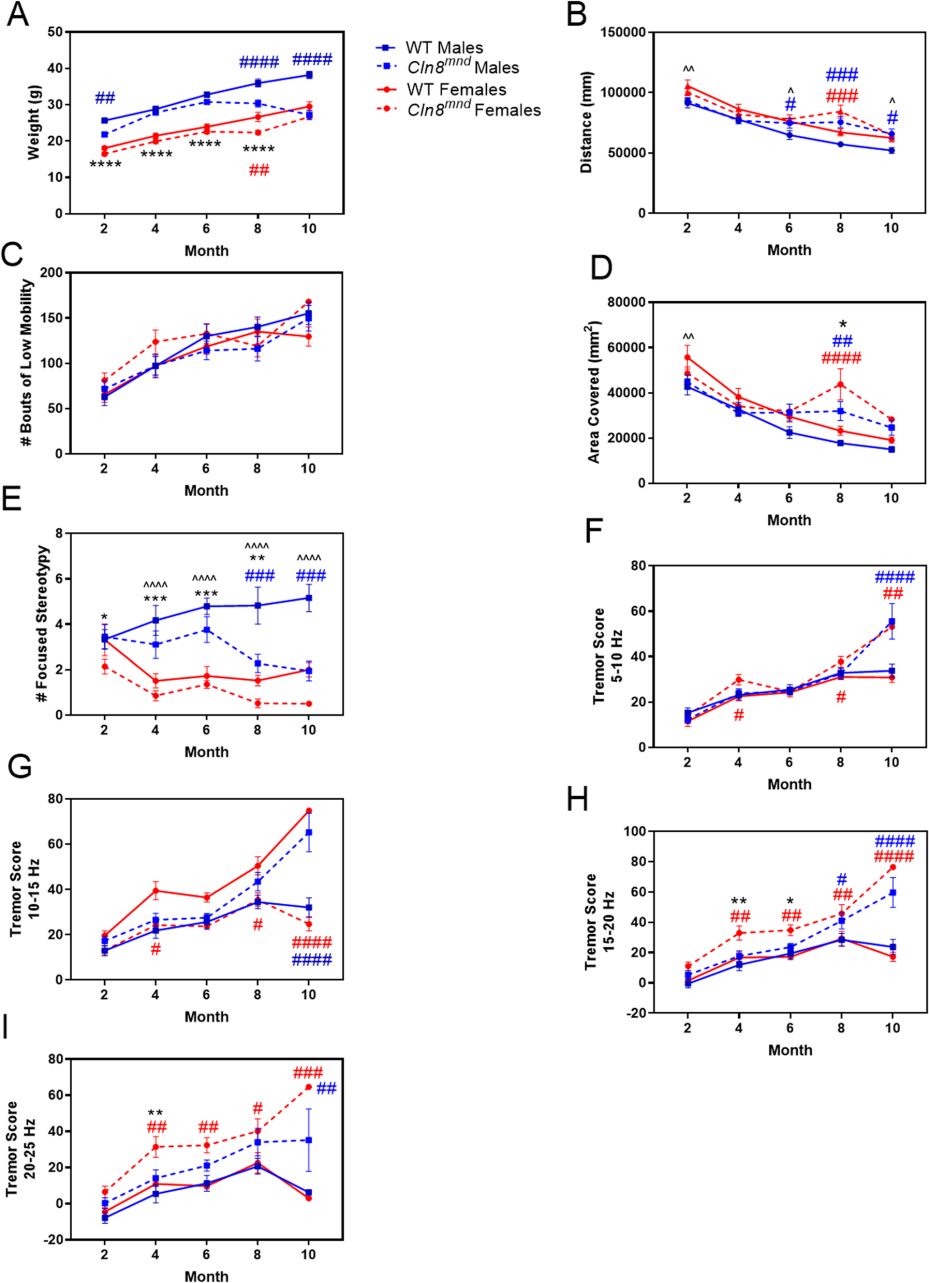
Sex differences in force plate actimeter results. *Cln8*^*mnd*^ males weighed significantly more than *Cln8*^*mnd*^ females at 2, 4, 6, and 8 months of age and started losing weight at 6 months of age (**A**). No consistent differences were observed in distance travelled (**B**), bouts of low mobility (**C**), or area covered (**D**). *Cln8*^*mnd*^ males exhibited greater frequency of focused stereotypy at 2, 4, 6, and 8 months of age (**E**). Comparisons of tremor scores revealed that *Cln8*^*mnd*^ females had higher tremor scores than their male counterparts at frequencies 15–20 Hz and 20–25 Hz (**F**–**I**). Comparisons of wild type males versus wild type females^^^, *Cln8*^*mnd*^ males versus *Cln8*^*mnd*^ females*, *Cln8*^*mnd*^ males versus wild type males^#^, and *Cln8*^*mnd*^ females versus wild type females^#^. Two-way ANOVA with Fisher’s LSD post-hoc. Mean ± SEM, n = 1–11 animals/sex/group, detailed n described in Additional file 4: Table S1. *p < 0.05, **p < 0.01, ***p < 0.001, ****p < 0.0001

Animals were also measured for general locomotor ability and tremor presence using a force plate actimeter. *Cln8*^*mnd*^ males began losing weight at 6 months of age while their female counterparts generally did not, though all *Cln8*^*mnd*^ animals were within healthy weight ranges for their sex (Fig. 4A). While there were some differences between genotypes and sexes in general activity (Fig. 4B–D; distance travelled, bouts of low mobility, and area covered), *Cln8*^*mnd*^ males consistently exhibited a greater number of focused stereotypies (i.e., rearing) as compared to *Cln8*^*mnd*^ females at 2, 4, 6, and 8 months of age (Fig. 4E). The same pattern was seen in wild type mice from 4, 6, 8, and 10 months of age, indicating this is likely related to male behavior as a whole. When assessing tremor presence, *Cln8*^*mnd*^ females showed increased tremor scores significantly earlier than their male counterparts at several frequencies, displaying increased tremors as early as 4 months of age while *Cln8*^*mnd*^ males showed tremors beginning at 8–10 months of age (Fig. 4F–I). Several other motor-behavioral tests were conducted, including an accelerating rotarod and vertical pole climb, and no sex dependent differences in *Cln8*^*mnd*^ mice were observed (Additional file 3: Fig. S3). Taken together, *Cln8*^*mnd*^ females consistently show a significantly faster and more severe disease progression than their male counterparts, including an earlier presence of tremors, earlier MWM deficits that are indicative of memory, learning, or visual deficits, and an earlier death.

### AAV9 gene therapy ameliorates disease pathogenesis and overall sex discrepancies

We recently published an investigation of a virally-delivered gene therapy vector (scAAV9.pT-MecP2.CLN8; ‘AAV9-CLN8’) in *Cln8*^*mnd*^ mice that demonstrated this therapeutic agent can improve lifespan and treat pathological and behavioral abnormalities in *Cln8*^*mnd*^ mice when delivered at postnatal day 1 via intracerebroventricular injection at 5.0 × 10^10^ vg/animal [6]. However, comparisons between sexes in response to therapy were not previously examined. Therefore, to determine if sex had an impact on AAV9-CLN8 treatment response, immunohistochemistry and behavioral data was examined across sexes in AAV9-CLN8 treated animals from 2 to 24 months of age.

**Fig. 5:**
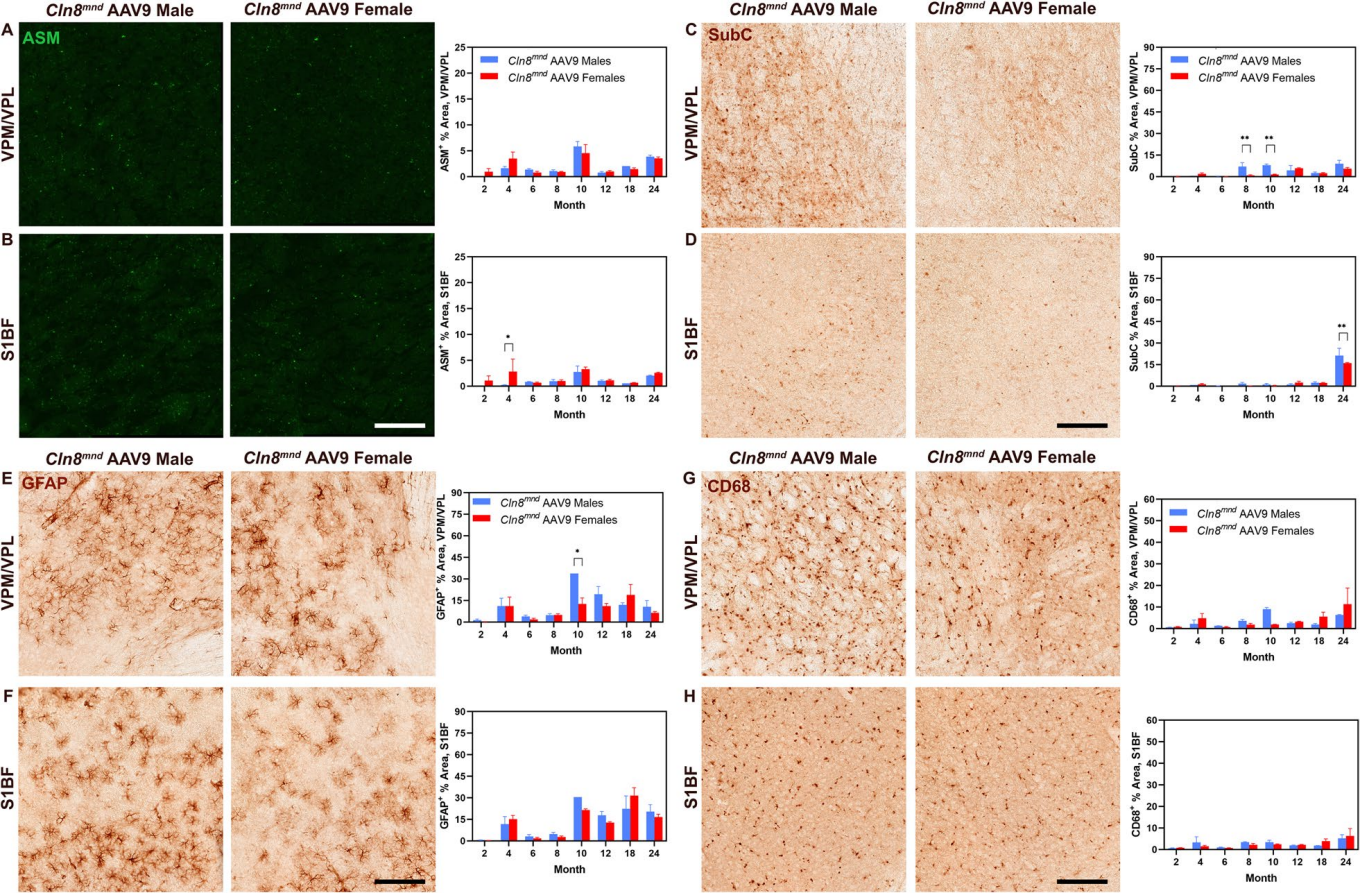
Sex-dependent histopathological differences in AAV9-treated *Cln8*^*mnd*^ mice. No sex-dependent histopathological differences were observed when comparing ASM accumulation in AAV9-treated *Cln8*^*mnd*^ males and females in the VPM/VPL (**A**). AAV9-treated *Cln8*^*mnd*^ females exhibit greater ASM accumulation within the S1BF at 4 months of age (**B**). AAV9-treated *Cln8*^*mnd*^ males evidenced enhanced SubC burden at 8 and 10 months of age within the VPM/VPL (**C**) and at 24 months of age within the S1BF (**D**). Greater astrocytosis (GFAP^+^) was observed in AAV9-treated *Cln8*^*mnd*^ males at 10 months of age within the VPM/VPL (**E**), but not within the S1BF (**F**). No sex-dependent differences observed in microgliosis (CD68^+^) in the VPM/VPL (**G**) or S1BF (**H**). Two-way ANOVA with Fisher’s LSD post-hoc. Mean ± SEM, n = 1–4 animals/sex/group, detailed n described in Additional file 4: Table S1. *p < 0.05, **p < 0.01

We previously described a robust reduction of ASM and SubC accumulation in *Cln8*^*mnd*^ animals treated with AAV9-CLN8 [6]. This reduction was most pronounced through 8 months of age, with slight increases in accumulation seen in treated animals from 10 to 24 months of age, though this accumulation did not reach the same burden as end-stage untreated *Cln8*^*mnd*^ mice. When split by sex, there were overall no differences to AAV9-CLN8 response to ASM prevention between the sexes (Fig. 5A, B). Treated *Cln8*^*mnd*^ males had increased SubC at 8, 10, and 24 months of age, although the general response was similar between the sexes (Fig. 5C, D).

In terms of glial reactivity, we previously reported significant attenuation of GFAP^+^ astrocytosis and CD68^+^ microgliosis in AAV9-CLN8 treated *Cln8*^*mnd*^ animals through 8 months of age [6]. From 10 to 24 months of age, however, both astrocytosis and microgliosis increased in AAV9-CLN8 treated animals, indicating a heightened and sustained inflammatory response. When analyzing the data by sex, there were no consistent differences in gliosis between the sexes of AAV9-CLN8 treated animals (Fig. 5E–H), indicating these heightened inflammatory responses are not sex-specific.

**Fig. 6:**
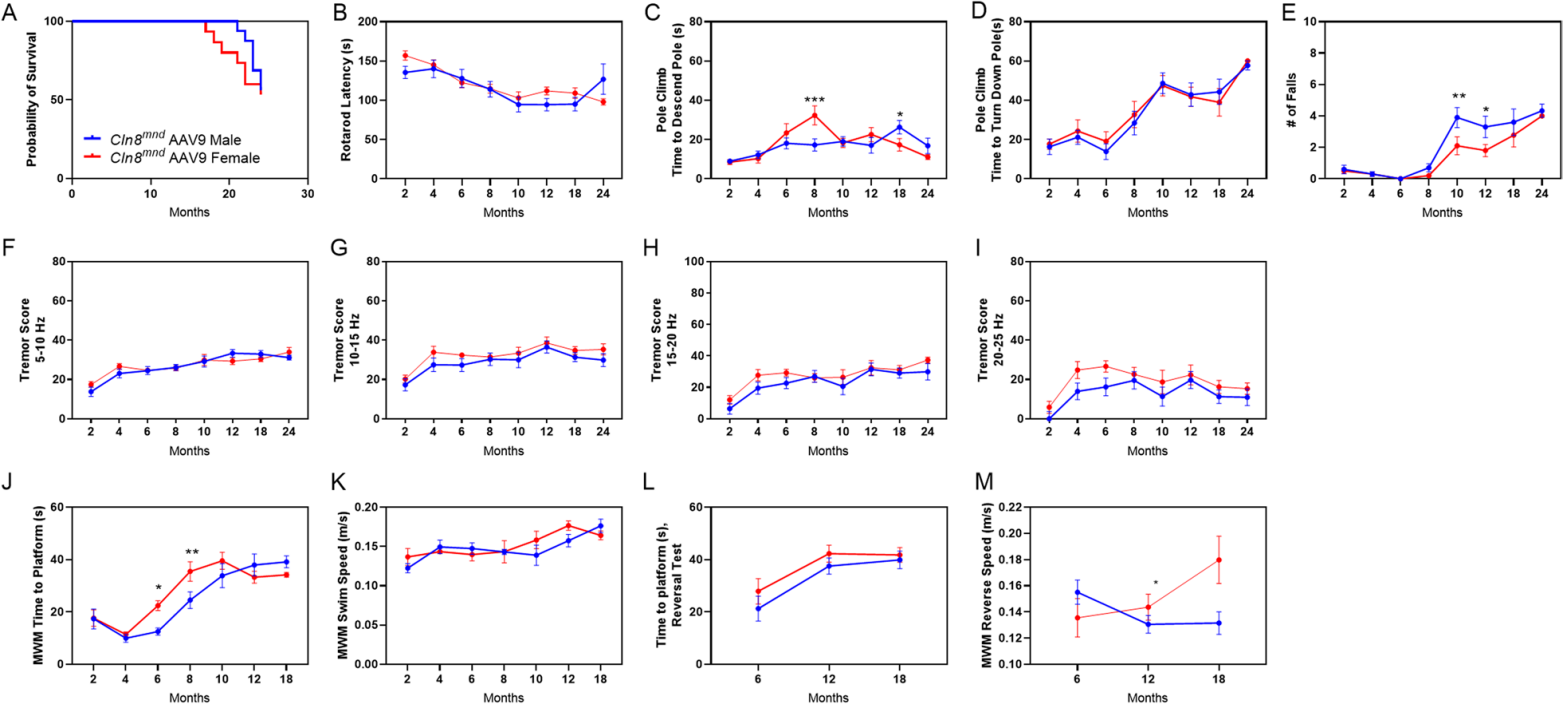
Analysis of sex-dependent differences in life span and motor-behavioral outcomes of AAV9-treated *Cln8*^*mnd*^ mice. AAV9-treated *Cln8*^*mnd*^ mice live similar lifespans regardless of sex (**A**). AAV9-treated *Cln8*^*mnd*^ mice perform similarly in an accelerated rotarod test (**B**). Transient sex-dependent differences detected at 8 and 18 months of age in the pole climb measurement “time to descend pole” (**C**), while no differences were detected in “time to turn down pole” (**D**), and differences detected at 10 and 12 months of age in “number of falls from the pole” (**E**). No differences detected in tremor presence between the sexes of AAV9-treated *Cln8*^*mnd*^ mice (**F**-**I**) *Cln8*^*mnd*^ females treated with AAV9-CLN8 were significantly slower at the Morris water maze (MWM) at 6 and 8 months of age (**J**), which was not accounted for by swim speed (**K**). No differences were observed in completion time of the reverse MWM (**L**) but AAV9-treated *Cln8*^*mnd*^ females had a higher swim velocity (**M**). Survival curve: log-rank (Mantel–Cox); n = 15–16 animals/sex. Two-way ANOVA with Fisher’s LSD post-hoc. Mean ± SEM, n = 5–11 animals/sex/group, detailed n described in Additional file 4: Table S1. *p < 0.05, **p < 0.01, ***p < 0.001, ****p < 0.0001

Lastly, we previously reported that AAV9-CLN8 treatment largely prevented behavioral deficits in *Cln8*^*mnd*^ animals, including preservation of motor abilities through 24 months of age (as measured by an accelerating rotarod and vertical pole climb), prevention of tremors through 12–18 months of age, and retention of a full lifespan of 24 months [6]. When examining these outcomes by sex, there were generally no differences between AAV9-CLN8 treatment response in lifespan, rotarod performance, performance in a vertical pole test, or tremor presence (Fig. 6A–I). A consistent difference was detected in the number of falls from the vertical pole test, where male AAV9-treated animals showed a slight but significant increase in falls when compared to female counterparts, though this resolved over time and was less than the fall frequency of untreated *Cln8*^*mnd*^ mice (Fig. 6E; Additional file 1: Fig. S1D). In the MWM, where we previously reported that AAV9-treated animals performed poorly at the task beginning at 6 months of age, sex-split analysis interestingly showed that AAV9-treated *Cln8*^*mnd*^ females were significantly longer to complete the task than their male counterparts at 6 and 8 months of age, (Fig. 6J, K). Surprisingly, this difference was not reflected in the reversal test and was not explained by swim speed (Fig. 6K–M). Taken together, there were scant differences in male and female response to AAV9-CLN8 gene therapy in pathology, behavior, and survival outcomes, and while AAV9-treated *Cln8*^*mnd*^ females experienced poorer MWM performance than their male counterparts early in disease course, it is unclear if this is due to an altered response to treatment or simply due to the trajectory of disease in a typical female animal.

## Discussion

This study demonstrates sex differences in the progression of CLN8 disease in the *Cln8*^*mnd*^ murine model. Specifically, female *Cln8*^*mnd*^ mice performed worse on the MWM assessment, perished earlier, and showed increased astrocyte and microglia reactivity over their *Cln8*^*mnd*^ male counterparts at several time points. Our reported results of ASM and SubC accumulation comparisons between *Cln8*^*mnd*^ male and female mice demonstrated contrasting data in that storage accumulation was more pronounced at different time stages of pathogenesis. Generally, *Cln8*^*mnd*^ male mice had greater ASM accumulation within the VPM/VPL and S1BF whereas *Cln8*^*mnd*^ female mice had greater SubC burden within both areas and the striatum. Accumulation is thought to occur due to any disruption in the basic processes of autophagy, lysosomal function, or oxidative damage; however, other mechanisms of accumulation may exist [38]. The primary storage material of ASM within CLN8-Batten disease is SubC, although, other disease subtypes may have a differing primary constituent like sphingolipid activator proteins in CLN1 and CLN10-Batten disease [38, 39]. Other ASM accumulation components include neutral lipids, phospholipids, dolichol pyrophosphate linked oligosaccharides, lipid linked oligosaccharides, dolichol esters, and metal ions [38, 40, 41]. Based on our data suggesting *Cln8*^*mnd*^ female mice having a greater SubC component of ASM, it is thus presumed their male comparisons are accumulating other molecular components from an unknown mechanism.

Interestingly, there was a marked increase in glial activity between 2 and 4 months of age, indicating this may be the critical time point in which pathological change from both these processes occurs. It is possible that this increase in gliosis may be a contributing factor to the poorer MWM performance and decreased lifespan seen within *Cln8*^*mnd*^ females. Previous investigations of neural injuries in mice offer support for an association between enhanced gliotic activity and poorer motor-behavioral outcomes in assessments like the MWM [42–44]. However, it is worth noting that prior investigation of sex differences of a CLN6 disease mouse model revealed *Cln6*^*nclf*^ males experience greater microgliosis than *Cln6*^*nclf*^ females at 6 months of age within the S1BF despite *Cln6*^*nclf*^ females perishing earlier and exhibiting poorer motor-behavioral outcomes [31]. These differences in pathological variations, such as increases in male ASM versus female SubC and increases in female gliosis in one NCL model versus male gliosis in another, highlight the complexity of interpreting pathological changes and their relation to disease progression and treatment outcomes, and specifically suggest that a more holistic approach may be required for this purpose. Unfortunately, these sex-dependent murine model differences cannot be correlated with clinical outcomes in humans with CLN8 disease since there have been no such detailed human investigations, likely due to small patient populations and the difficulty in comparing human subjects due to environmental differences and genetic heterogeneity of *CLN8* mutations [45].

Greater pathological visual deficits and/or dysfunction are another possible explanation for worse MWM performance by *Cln8*^*mnd*^ females. *Cln8*^*mnd*^ females previously demonstrated harsher retinal histopathologic profiles and retina cell apoptosis compared to *Cln8*^*mnd*^ male comparisons [32], and we hypothesize these differences and increased activated glia may contribute to *Cln8*^*mnd*^ females’ greater visual aberrations and poorer performance [46, 47]. Prior investigations have highlighted glial dysfunction in NCL murine models coinciding with subsequent neuronal damage of the visual cortex and retina, resulting in deterioration of visual perception and retinal function [48–50]. Moreover, attenuation of inflammatory microglia via therapeutic agents in Batten disease animal models improved visual acuity, reduced retinal thinning, and improved retinal ganglion cell survival [49, 51–53]. Sex comparisons of microglia contribution to pathology and response to therapy in vision related systems may better elucidate this process.

An increasing body of evidence indicates that aberrant glial cell function contributes to the disruption of CNS homeostasis and resulting neurodegeneration in Batten disease [54, 55]. Broadly, activation of astrocytes and microglia predicts subsequent neuron degeneration within the local area in various Batten disease models, and in the *Cln8*^*mnd*^ mouse model specifically, enhanced gliosis coincides with further disease progression [10, 56]. More recently, investigation of in vitro glial cultures derived from CLN1 and CLN3 murine models demonstrates the negative influence of glia on neuron survival through differing phenotypic functional states [57–59]. *Ppt1*^−*/*−^ microglia cultures were shown to exist in a basally activated state with increased secretion of cytokines and chemokines that induce neuron death, and similarly, cultured *Cln3*^*Δex7*/8^ microglia behave in a reactionary state where stimuli elicit a caspase-1 mediated pro-inflammatory response that includes cytokine/chemokine production, glutamate release, and hemichannel activity that induces cell death [57, 59]. Furthermore, depletion of microglia via pharmacologic targeting can improve CLN1 disease in mice, and interestingly, Berve et al. observed surprising sex and anatomical region biases: greater preservation of *Ppt1*^−/−^ female microglia was observed as they were less responsive to pharmacologic treatment, especially within the S1BF, and females experienced subsequently poorer treatment outcomes compared to their male counterparts [51].

Nonetheless, the question remains why *Cln8*^*mnd*^ females exhibit enhanced microglial activation within the S1BF and VPM/VPL nuclei of the thalamus. Within murine brains, sexual dimorphism has been noted in microglia function, morphology, and colonization of brain structures–stemming from variance in sex-specific gene expression, circulating sex steroidal hormones and response to hormones, and epigenetic interactions [60–63]. Female-derived mouse microglia tend to be more reactive and inflammatory than male-derived microglia, characterized by higher inflammatory cytokines, inflammatory-related receptor expression, and differential expression of estrogen receptor subtypes [61]. Comparison of microglial number within the amygdala, hippocampus, and parietal cortex revealed that male mice had more microglia in the initial post-natal period, coinciding with their testosterone surge, until the transition into adolescence when females exhibited greater microglia with an activated phenotype in the same regions [60]. The sex differences in microglial colonization may be influenced by disparate levels of sex hormones and chemokines, as evidenced by a 200-fold influx of CCL20 and 50 fold increase of CCL4 during the testosterone surge in early male mouse development [60, 64, 65]. Therefore, sex-dependent chemokine expression in *Cln8*^*mnd*^ mice is a possible explanation for the relatively increased microgliosis observed in *Cln8*^*mnd*^ females at later life stages, and should be investigated further.

Sexual dimorphism in genetic architecture and X-chromosome gene regulation may promote the chronic inflammatory process in Batten disease, and thus may partially explain the exacerbated phenotype observed within females [66–69]. The X-chromosome is the locus of numerous genes related to immune function and regulation and through mechanisms like mosaic X-chromosome inactivation and “gene escape” from the inactivated X-chromosome, may lead to differential and bi-allelic expression of proinflammatory genes respectively [67, 68, 70, 71]. An estimated 3–7% and 15–23% of genes on the inactivated X-chromosome escape in female mice and humans respectively [70, 72, 73]. For example, cluster of differentiation (CD) 40 and 99 ligand are expressed on the X-chromosome and increased CD receptor-CD ligand engagement activates proinflammatory cascades involving T and B cells, monocyte derivatives like macrophages and microglia, and cytokine upregulation which is implicated in a multitude of neurologic disease [74, 75]. To our knowledge, no such studies have investigated the degree to which X-chromosome inactivation escape may influence the poorer histopathologic and motor-behavioral outcomes observed within female sex in Batten disease. Elucidation of the likely mechanism(s) by which this process occurs may provide insight for potential therapeutic targets to alleviate disease burden.

We also reported that AAV9 gene therapy was well received and generally efficacious to the same degree in *Cln8*^*mnd*^ mice regardless of sex, with one exception where AAV9-treated female mice performed worse on MWM assessments than their male counterparts, which as discussed may be due to the relatively worse retinal damage experienced by *Cln8*^*mnd*^ females [32]. There have been few publications on sexual dimorphism in AAV-mediated gene therapy, though reports have indicated differences in tissue transduction depending on serotype, route of administration, tissue type, and the presence of single or double-stranded genomes, with the most commonly affected tissues being the liver, skeletal muscle, and gonads [76–78]. Specifically, one detailed report described how male-specific increases in liver transduction were the result of androgen-dependent pathways, and that modulating these pathways led to improved transduction in the livers of female mice [79]. While there is limited data on sex-dependent differences of AAV-gene therapy in the CNS, one recent study demonstrated sex-specific responses to intracerebroventricularly delivered AAV9 in a mouse model of Dravet syndrome, a debilitating seizure disorder caused by mutations in the α subunit of NaV1.1 channels (*SCN1A*) [80]. The authors speculated that these sex-specific differences occurred due to basal differences in voltage-gated sodium channel presence in male and female mice, indicating that any sex-specific differences in response to gene therapies, or lack of differences, may be due to whether there are sexually dimorphic functions already present for the protein product in question.

## Conclusions

Taken together, the results from this investigation provide further evidence of sex-dependent differences in lifespan, histopathology, and motor-behavioral outcomes within the *Cln8*^*mnd*^ mouse model of Batten disease, and gives insight into sex-dependent responses to CNS-delivered AAV9 gene therapy. Although sex discrepancies have been observed in human subjects with CLN3-Batten disease, sparse information exists for other forms of NCLs. As such, based on the surmounting body of evidence demonstrating the importance of sex as a biologic modifier, prospective and retrospective analysis of sex differences in other forms of Batten disease should be conducted to yield a better understanding of disease pathogenesis and treatment response.

## Materials and methods

The majority of the data presented in this manuscript was previously published as a mixed-sex cohort in Johnson et al. [6] where the authors showed AAV9-gene therapy of CLN8 prevented CLN8 Batten disease characteristics within *Cln8*^*mnd*^ mice. The present manuscript primarily examines that previously collected data as a sex-split cohort, and adds additional analyses not previously published.

### Ethics statement/animals

Wild type and homozygous *Cln8*^*mnd*^ mice on C57BL/6J backgrounds were used for all studies and were housed under identical conditions in an AAALAC accredited facility in accordance with IACUC approval (Protocol #: 178-02-24D Sanford Research, Sioux Falls, SD). Animals were bred from standing colonies at Sanford Research. *Cln8*^*mnd*^ animals exhibit a single nucleotide insertion (267–268C, codon 90) predicting a premature termination codon. Wild type animals lacked this mutation.

### AAV9-treatment

*Cln8*^*mnd*^ mice were treated with scAAV9.pT-MecP2.CLN8 via intracerebral ventricular injection (ICV) on postnatal day 1 as previously described at a dose of 5.0 × 10^10^ vg/animal [6].

### Immunohistochemistry

Mice were CO_2_ euthanized, cardiac perfused with phosphate-buffered saline, and the left hemisphere of the brain fixed in 4% paraformaldehyde. The brain was sectioned on a vibratome into 50 μm slices and immunohistochemistry was performed on free-floating sections as previously described using anti-ATP synthase subunit C (Abcam, ab181243), anti-GFAP (Dako, Z0334), and anti-CD68 (AbD Serotec, MCA1957) antibodies [6]. Secondary antibodies included anti-rat and anti-rabbit biotinylated (Vector Labs, BA-9400). Sections were imaged and analyzed using an Aperio Digital Pathology Slide Scanner (VERSA) and associated software. Regions of interest were extracted in triplicate and subdivided into 4 quadrants for analysis. Immunolabeling was quantified using ImageJ.

ASM data was collected by methods previously described [6] with right hemisphere placed on a 1 mm sagittal brain block. Tissue blocks from 0 to 3 mm right of the midline were flash-frozen, brain sections sliced on a cryostat at 16 μm, and placed on slides. Slides were briefly post-fixed in 10% NBF and series dehydrated, with nuclei labeled using DAPI and coverslips applied using antifade mounting media (Dako Faramount, Agilent Technologies). Sections were imaged using a Nikon fluorescent microscope and quanitified using ImageJ.

Cortical thickness measurements were obtained in the motor and somatosensory cortex of coronal tissue sections labeled with nuclear dye. Measurements were taken as previously described [31], as triplicates of the cortical plate encompassing layers 1–6 of the cerebral cortex.

### Neurobehavior testing

#### Rotarod

Animals participated in an accelerating Rotarod protocol as previously described to assess motor coordination (Columbus Instruments, Columbus, OH, USA) [6]. The machine was set to accelerate 0.3 rpm every two seconds, with a starting speed of 0.3 rpm and a maximum speed of 36 rpm. Briefly, mice were trained for nine trials in the morning (3 sets of three consecutive trials followed by a 30 min rest), given a four-hour rest period, and tested in nine trials in the afternoon (3 sets of three consecutive trials followed by a 30 min rest). The latency to fall from the rod (time in seconds) was averaged from each of the nine afternoon testing sessions to produce one value per mouse.

#### Pole climb

The pole climb descent test was performed as previously described [6]. Mice were placed downward on a metal pole for 5 trials and given 60 s to descend the pole each trial. Mice were then placed upward on a metal pole for 4 trials and given 60 s to turn downward on the pole for each trial. Lastly, the number of falls made by each mouse during the 9 trials was recorded.

#### Water maze

Mice were tested in a 4 foot diameter Morris Water Maze apparatus as previously described [6]. Briefly, the apparatus was filled with water to ~ 26 inches, the goal platform submerged by 0.5 cm at 315°, and the tub aligned with four distinct visual cues at 0, 90, 180, and 270° to aid in spatial memory. After mice were trained in a clear pool with a flagged platform, mice were trained to find a hidden platform in opaque water over four trials in the morning (60 s consecutive trials). Mice were then given a three-hour rest period and tested over four trials in the afternoon (60 s consecutive trials). Mice were tested for four consecutive days, each day starting at a different visual cue. Mice were recorded using Any-maze video tracking software (Stoelting Co., Wood Dale, IL, USA), and test duration and swim speed were averaged from the sixteen afternoon trials performed by each mouse.

#### Clasping, ledge, and gait tests

Tests were performed as previously described [6]. For hind limb clasping measurements, animals were scored on the extent to which their limbs clasped into their abdomen when held by the base of their tail (score 0–3). For ledge lowering measurements, animals were scored on their ability to climb down from the edge of their home cage (score 0–3). For gait measurements, animals were scored on their overall ease of walking, including whether their abdomen dragged on the ground and if their limbs were splayed out while walking (score 0–3). The scores were examined as individual tests and collectively as a score from 0 to 9. The same blinded experimenter determined all scores.

#### Force plate

A force plate actimeter was used to measure locomotion and tremors as previously described [6]. Animals were recorded in a sound-proof chamber for 20 min and data was processed using FPA Analysis Software (BASi, West Lafayette, IN) (Additional file 3: Fig. S3).

### Statistical analysis

Statistical analyses were performed using GraphPad Prism (v9.0.2 or equivalent) and details are noted in the figure legends. In general, a two-way ANOVA was employed with Fisher’s LSD, and outliers were removed with the ROUT method, Q = 0.1%. If appropriate, an unpaired t-test was used. For the survival curve analysis, the log-rank (Mantel–Cox) test was used. *p < 0.05, **p < 0.01, ***p < 0.001, ****p < 0.0001. Detailed sample n’s are described in Additional file 4 Table S1.

The data utilized within this study was previously published by Johnson et al. as a combined sex dataset only [6], and the current study expands on this data by doing an in-depth sex split analysis.

## Supplementary Information

**Additional file1**. **Figure S1**: Female Cln8mnd mice show enhanced subunit c accumulation and glial activation in the striatum and hippocampus. Cln8mnd females show enhanced SubC accumulation at 8 months within the striatum, while no sex differences are detected in the CA3 region of the hippocampus (A). Female Cln8mnd mice have greater GFAP+ astrocyte expression within the striatum at 8 months of age, but not in the CA3 (B). Female Cln8mnd mice exhibit enhanced microglial activation (CD68+) at 8 months of age within the striatum and CA3 of the hippocampus (C). Two-way ANOVA with Fisher’s LSD post-hoc. Mean ± SEM, n=2-3 animals/sex/group, detailed n described in Additional file 4: Table S1. *p<0.05, **p<0.01, ***p<0.001, ****p<0.0001. Scale Bars: 150 µm.

**Additional file2**. **Figure S2**: Cln8mnd mice show no thinning of the cerebral cortex at 2 and 6 months of age.

**Additional file3**. **Figure S3**: Comparison of wild type and Cln8mnd mice on rotarod and pole climb assessments. Cln8mnd animals perform poorly in an accelerating rotarod test by 6 months of age, with Cln8mnd animals performing similarly regardless of sex (A). Cln8mnd animals perform poorly in pole climb assessment by 8 months of age regardless of sex (B-D). Comparisons of wild type males vs. wild type females^, Cln8mnd males vs. Cln8mnd females*, Cln8mnd males vs. wild type males#, and Cln8mnd females vs. wild type females#. Two-way ANOVA with Fisher’s LSD post-hoc. Mean ± SEM, n=1-11 animals/sex/group, detailed n described in Additional file 4: Table S1. *p<0.05, **p<0.01, ***p<0.001, ****p<0.0001.

**Additional file4**. **Table S1**: Detailed animal n for each experiment (n=number of animals; Male/Female).

## Data Availability

The datasets used and/or analyzed during the current study are available from the corresponding author on reasonable request.

## Abbreviations

NCL(s): Neuronal ceroid lipofuscinoses
vLINCL: Variant late-infantile neuronal ceroid lipofuscinoses
JNCL: Juvenile neuronal ceroid lipofuscinoses
CLN (1, 2, 3, 6, 8, 10): Ceroid lipofuscinosis neuronal genes, designated as CLN1, CLN2, CLN3, CLN6, CLN8, CLN10, etc.
*Cln8*^*mnd*^: Mouse model of CLN8 disease
*Cln6*^*nclf*^: Mouse model of CLN6 disease
*Cln3*^*Δex7/8*^: Mouse model of CLN3 disease
ER: Endoplasmic reticulum
CNS: Central nervous system
VPM/VPL: Ventral posteromedial/ventral posterolateral nuclei of the thalamus
S1BF: Somatosensory cortex, barrel field
CA3: Cornu ammonis 3, region of the hippocampus
ASM: Autofluorescent storage material
SubC: Mitochondrial atp synthase subunit c
GFAP: Glial fibrillary acidic protein
CD68, CD40, CD99: Cluster of differentiation protein 68; 40; 99
CCL4, CCL20: Chemokine ligand 4; 20
SCN1A: Sodium channel protein type 1 subunit alpha
CO_2_: Carbon dioxide
NBF: Neutral buffered formalin
PBS: Phosphate buffered saline
DAPI: 4′,6-Diamidino-2-phenylindole
AAV9: Adeno associated virus, serotype 9
scAAV9.pT-MecP2.CLN8 (AAV9-CLN8): Self-complimentary adeno associated virus serotype 9, targeting CLN8 with a truncated methyl-CpG-binding protein promoter. Designated as AAV9-CLN8
ICV: Intracerebroventricular
Vg: Viral genomes
MWM: Morris water maze
RPM: Rotations per minute
FPA: Force plate actimeter
ANOVA: Analysis of variance
LSD: Least significant difference
ROUT: Robust regression and outlier removal
SEM: Standard error of the mean
Hz: Hertz
AAALAC: Association for Assessment and Accreditation of Laboratory Animal Care
IACUC: Institutional Animal Care and Use Committee
NIH: National Institutes of Health
