# Supplementary figures and images for: Sex-split analysis of pathology and motor-behavioral outcomes in a mouse model of CLN8-Batten disease reveals an increased disease burden and trajectory in female Cln8mnd mice
Source: Orphanet J Rare Dis. 2022 Nov 11;17:411. doi: 10.1186/s13023-022-02564-7 (PMC9652919; doi:10.1186/s13023-022-02564-7)

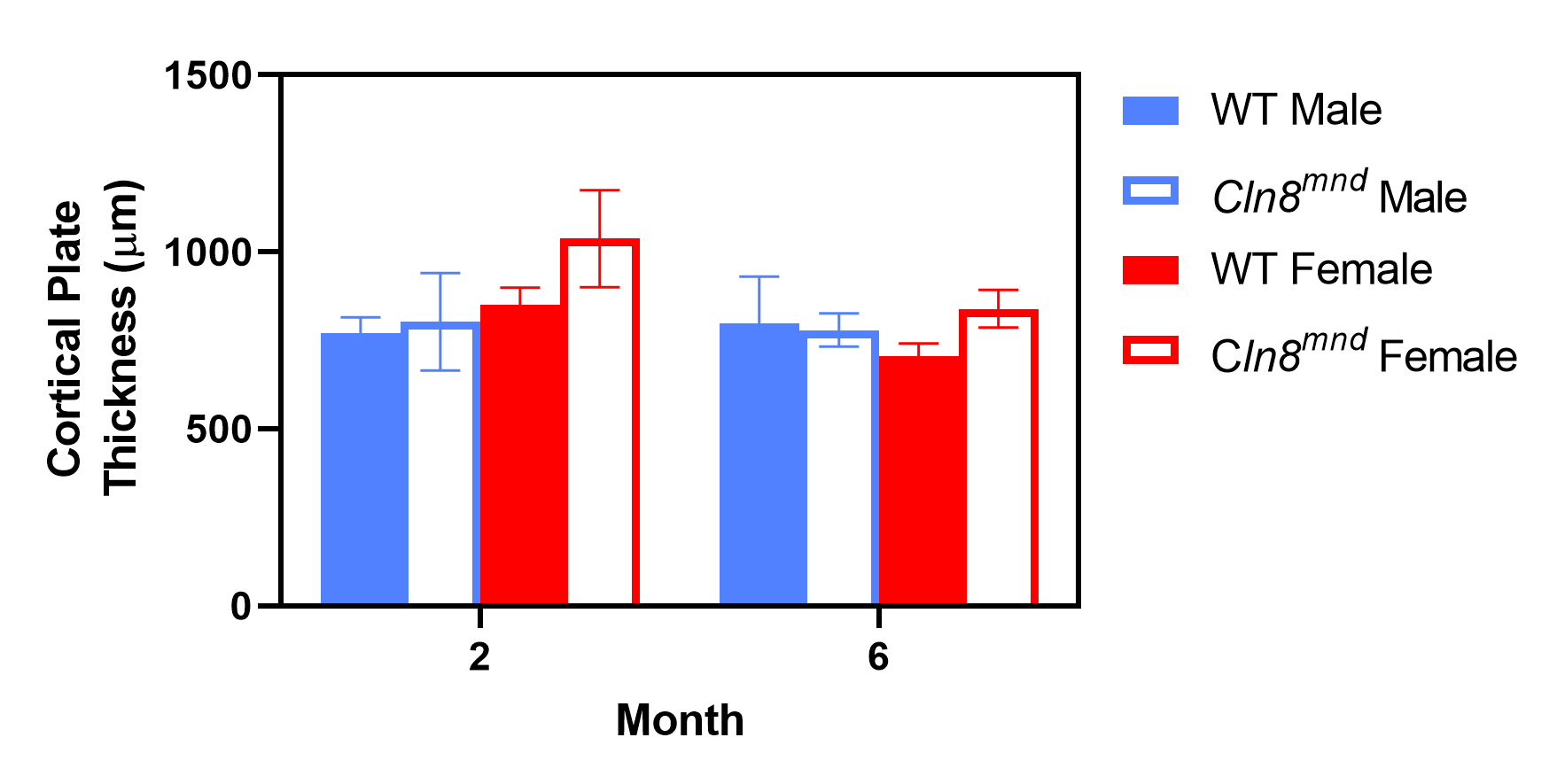

Supplement: Supplementary file 2 — Additional file2. Figure S2: Cln8mnd mice show no thinning of the cerebral cortex at 2 and 6 months of age. [file 13023_2022_2564_MOESM2_ESM.tif]

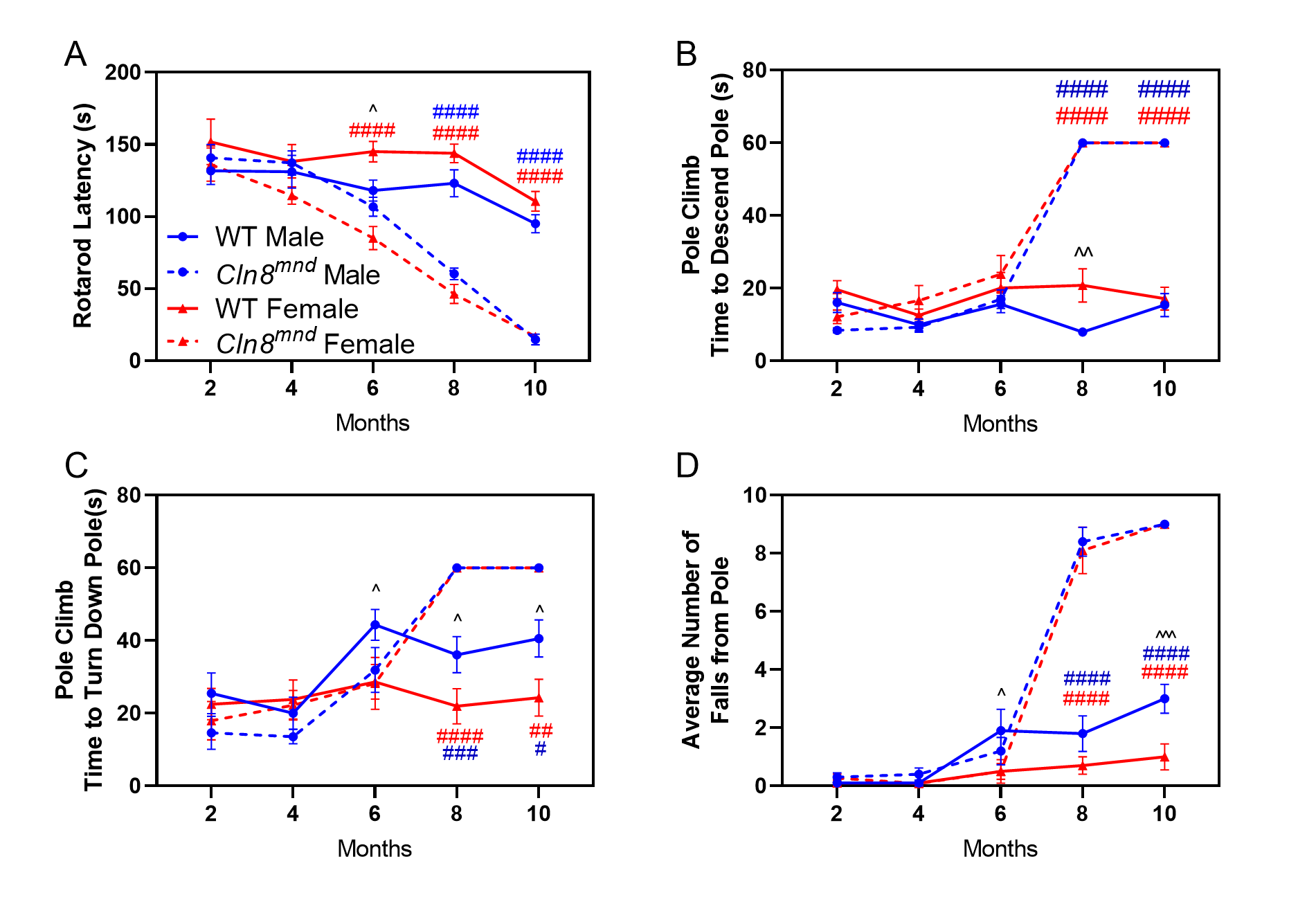

Supplement: Supplementary file 3 — Additional file3. Figure S3: Comparison of wild type and Cln8mnd mice on rotarod and pole climb assessments. Cln8mnd animals perform poorly in an accelerating rotarod test by 6 months of age, with Cln8mnd animals performing similarly regardless of sex (A). Cln8mnd animals perform poorly in pole climb assessment by 8 months of age regardless of sex (B-D). Comparisons of wild type males vs. wild type females^, Cln8mnd males vs. Cln8mnd females*, Cln8mnd males vs. wild type males#, and Cln8mnd females vs. wild type females#. Two-way ANOVA with Fisher’s LSD post-hoc. Mean ± SEM, n=1-11 animals/sex/group, detailed n described in Additional file 4: Table S1. *p<0.05, **p<0.01, ***p<0.001, ****p<0.0001. [file 13023_2022_2564_MOESM3_ESM.tif]
