# Supplementary material for: Sex-split analysis of pathology and motor-behavioral outcomes in a mouse model of CLN8-Batten disease reveals an increased disease burden and trajectory in female Cln8mnd mice
Source: Orphanet J Rare Dis. 2022 Nov 11;17:411. doi: 10.1186/s13023-022-02564-7 (PMC9652919; doi:10.1186/s13023-022-02564-7)
Supplement: Supplementary file 4 — Additional file4. Table S1: Detailed animal n for each experiment (n=number of animals; Male/Female). [file 13023_2022_2564_MOESM4_ESM.docx]

**Supplemental Table 1. Detailed animal n for each experiment (n=number of animals; Male/Female).**

| n=# of animals; Male/Female | | WT | | | | | *Cln8^mnd^* | | | | | *Cln8^mnd^* +AAV9 | | | | | | | |
| --- | --- | --- | --- | --- | --- | --- | --- | --- | --- | --- | --- | --- | --- | --- | --- | --- | --- | --- | --- |
|  |  | 2 | 4 | 6 | 8 | 10 | 2 | 4 | 6 | 8 | 10 | 2 | 4 | 6 | 8 | 10 | 12 | 18 | 24 |
| ASM | S1BF | 2/2 | 3/3 | 3/3 | 3/3 | - | 3/3 | 3/3 | 3/3 | 3/3 | - | 3/3 | 3/3 | 3/3 | 3/3 | 3/4 | 3/3 | 1/4 | 3/4 |
|  | VPM/VPL | 2/2 | 3/3 | 3/3 | 3/3 | - | 3/3 | 3/3 | 3/3 | 3/3 | - | 3/3 | 3/3 | 3/3 | 3/3 | 3/4 | 3/3 | 1/4 | 3/4 |
| SubC | S1BF | 3/2 | 3/3 | 3/3 | 3/3 | - | 2/1 | 3/2 | 3/3 | 3/3 | - | 1/3 | 1/3 | 3/3 | 3/3 | 3/2 | 3/3 | 2/4 | 2/4 |
|  | VPM/VPL | 3/2 | 3/3 | 3/3 | 3/3 | - | 2/2 | 3/2 | 3/3 | 3/3 | - | 1/3 | 1/3 | 3/3 | 3/3 | 3/2 | 3/3 | 2/4 | 2/4 |
|  | Striatum | - | - | 3/3 | 3/3 | - | - | - | 3/3 | 2/3 | - | - | - | - | - | - | - | - | - |
|  | CA3, Hipp | - | - | 3/3 | 3/3 | - | - | - | 3/2 | 2/2 | - | - | - | - | - | - | - | - | - |
| GFAP | S1BF | 3/3 | 3/3 | 3/3 | 3/3 | - | 3/3 | 3/3 | 3/3 | 3/3 | - | 2/3 | 3/3 | 3/3 | 3/3 | 1/2 | 3/3 | 2/4 | 2/4 |
|  | VPM/VPL | 3/3 | 3/3 | 3/3 | 3/3 | - | 3/2 | 3/3 | 3/3 | 3/3 | - | 2/3 | 3/3 | 3/3 | 3/3 | 1/2 | 3/3 | 2/4 | 2/4 |
|  | Striatum | - | - | 3/2 | 3/3 | - | - | - | 3/3 | 3/3 | - | - | - | - | - | - | - | - | - |
|  | CA3, Hipp | - | - | 2/3 | 3/3 | - | - | - | 3/3 | 3/3 | - | - | - | - | - | - | - | - | - |
| CD68 | S1BF | 3/3 | 3/3 | 3/3 | 3/3 | - | 3/3 | 2/3 | 3/3 | 3/3 | - | 2/3 | 2/3 | 3/3 | 3/3 | 3/2 | 3/3 | 2/4 | 2/4 |
|  | VPM/VPL | 3/3 | 3/3 | 3/3 | 3/3 | - | 3/3 | 2/3 | 3/3 | 3/3 | - | 2/3 | 2/3 | 3/3 | 3/3 | 3/2 | 3/3 | 2/4 | 2/4 |
|  | Striatum | - | - | 3/3 | 3/3 | - | - | - | 3/3 | 3/3 | - | - | - | - | - | - | - | - | - |
|  | CA3, Hipp | - | - | 2/3 | 3/3 | - | - | - | 3/3 | 3/3 | - | - | - | - | - | - | - | - | - |
|  | Cortical Thickness | 3/3 | - | 3/2 | - | - | 3/3 | - | 3/3 | - | - | - | - | - | - | - | - | - | - |
| Misc Behavior | Pole Climb: Climb Down | 10/10 | 9/10 | 10/10 | 8/10 | 10/10 | 9/10 | 8/11 | 10/10 | 8/9 | 6/2 | 10/9 | 10/9 | 10/10 | 10/10 | 10/10 | 10/10 | 10/9 | 6/6 |
|  | Pole Climb: Turn Down | 10/10 | 10/10 | 10/10 | 10/10 | 10/10 | 10/11 | 9/10 | 10/10 | 10/9 | 6/2 | 10/10 | 10/10 | 10/10 | 10/10 | 10/10 | 10/10 | 10/9 | 6/6 |
|  | Pole Climb: # Falls | 10/10 | 10/10 | 10/10 | 10/10 | 10/10 | 10/11 | 10/10 | 10/10 | 10/10 | 6/2 | 10/10 | 10/10 | 9/10 | 10/10 | 10/10 | 10/10 | 10/9 | 6/6 |
|  | Rotarod | 10/10 | 10/10 | 10/9 | 10/10 | 10/10 | 10/11 | 10/11 | 10/10 | 10/10 | 4/1 | 10/10 | 10/10 | 10/10 | 10/10 | 10/10 | 10/10 | 10/10 | 6/5 |
|  | Survival | 13/15 | | | | | 16/14 | | | | | 16/15 | | | | | | | |
| MWM | Time | 11/9 | 10/10 | 9/10 | 10/9 | - | 9/8 | 9/7 | 9/7 | 8/2 | - | 7/8 | 7/8 | 7/8 | 7/8 | 6/6 | 6/4 | 6/3 | - |
|  | Swim Speed | 11/9 | 10/10 | 10/9 | 10/10 | - | 9/8 | 9/7 | 9/7 | 8/2 | - | 7/8 | 7/8 | 7/8 | 7/8 | 6/6 | 6/4 | 6/3 | - |
|  | Reversal: Time | - | - | 9/10 | - | - | - | - | 9/7 | - | - | - | - | 7/8 | - | - | 6/4 | 6/3 | - |
|  | Reversal: Swim Speed | - | - | 10/10 | - | - | - | - | 9/7 | - | - | - | - | 7/8 | - | - | 6/4 | 6/3 | - |
| Force Plate | Weight | 8/10 | 10/10 | 10/10 | 10/10 | 10/10 | 10/10 | 10/11 | 10/11 | 10/10 | 5/1 | 11/9 | 10/9 | 10/10 | 10/10 | 10/10 | 10/10 | 10/10 | 6/6 |
|  | Total Distance | 9/10 | 10/10 | 10/10 | 10/10 | 10/10 | 10/10 | 10/11 | 10/11 | 10/10 | 5/1 | 11/9 | 10/10 | 10/10 | 10/10 | 10/9 | 10/10 | 10/10 | 6/6 |
|  | Total Area | 9/10 | 10/10 | 10/10 | 10/10 | 10/9 | 10/10 | 10/11 | 10/11 | 10/10 | 5/1 | 11/9 | 10/10 | 10/9 | 8/10 | 10/9 | 10/10 | 10/10 | 6/6 |
|  | Bouts Low Mobility | 9/10 | 10/10 | 10/10 | 10/10 | 10/10 | 10/10 | 10/11 | 10/11 | 10/10 | 5/1 | 11/9 | 10/10 | 10/10 | 10/10 | 10/10 | 10/10 | 10/10 | 6/6 |
|  | Stereotypy | 9/10 | 10/10 | 9/10 | 10/10 | 10/10 | 10/10 | 10/11 | 10/11 | 10/10 | 5/1 | 11/9 | 9/10 | 10/10 | 10/10 | 10/10 | 10/9 | 10/9 | 6/6 |
|  | Tremor Score (5-10Hz) | 10/10 | 10/10 | 10/10 | 10/10 | 10/10 | 10/11 | 10/11 | 10/11 | 10/10 | 5/1 | 10/10 | 10/10 | 10/10 | 10/10 | 10/10 | 10/10 | 10/10 | 6/6 |
|  | Tremor Score (10-15Hz) | 10/10 | 10/10 | 10/9 | 10/10 | 10/10 | 10/11 | 10/11 | 10/11 | 10/11 | 5/1 | 10/10 | 10/10 | 10/10 | 10/10 | 10/10 | 10/10 | 10/10 | 6/6 |
|  | Tremor Score (15-20Hz) | 10/10 | 10/10 | 10/9 | 10/10 | 10/10 | 10/11 | 10/11 | 10/11 | 10/10 | 5/1 | 10/10 | 10/10 | 10/10 | 10/10 | 10/10 | 10/10 | 10/10 | 6/6 |
|  | Tremor Score (20-25Hz) | 10/10 | 10/10 | 10/9 | 10/10 | 8/8 | 10/11 | 10/11 | 10/11 | 10/10 | 5/1 | 10/10 | 10/10 | 10/10 | 10/10 | 10/10 | 10/10 | 10/10 | 6/6 |
